# Organic Matter Detection on Mars by Pyrolysis-FTIR: An Analysis of Sensitivity and Mineral Matrix Effects

**DOI:** 10.1089/ast.2016.1485

**Published:** 2016-11-01

**Authors:** Peter R. Gordon, Mark A. Sephton

**Affiliations:** Impacts and Astromaterials Research Centre, Department of Earth Science and Engineering, Imperial College London, London, UK.

## Abstract

Returning samples from Mars will require an effective method to assess and select the highest-priority geological materials. The ideal instrument for sample triage would be simple in operation, limited in its demand for resources, and rich in produced diagnostic information. Pyrolysis–Fourier infrared spectroscopy (pyrolysis-FTIR) is a potentially attractive triage instrument that considers both the past habitability of the sample depositional environment and the presence of organic matter that may reflect actual habitation. An important consideration for triage protocols is the sensitivity of the instrumental method. Experimental data indicate pyrolysis-FTIR sensitivities for organic matter at the tens of parts per million level. The mineral matrix in which the organic matter is hosted also has an influence on organic detection. To provide an insight into matrix effects, we mixed well-characterized organic matter with a variety of dry minerals, to represent the various inorganic matrices of Mars samples, prior to analysis. During pyrolysis-FTIR, serpentinites analogous to those on Mars indicative of the Phyllocian Era led to no negative effects on organic matter detection; sulfates analogous to those of the Theiikian Era led, in some instances, to the combustion of organic matter; and palagonites, which may represent samples from the Siderikian Era, led, in some instances, to the chlorination of organic matter. Any negative consequences brought about by these mineral effects can be mitigated by the correct choice of thermal extraction temperature. Our results offer an improved understanding of how pyrolysis-FTIR can perform during sample triage on Mars. Key Words: Mars—Life-detection instruments—Search for Mars’ organics—Biosignatures. Astrobiology 16, 831–845.

## 1. Introduction

The search for extraterrestrial life within our solar system, extant or otherwise, represents a major goal of astrobiology. Mars presents itself as the best candidate for contemporary life search missions because it is the most Earth-like planet and is relatively accessible by spacecraft on reasonable timescales. Previous missions to Mars have been unable to detect evidence of past life, but their results have bolstered the case for further investigation. Evidence has accumulated that suggests Mars was warm and wet enough during parts of its history to be habitable by life (Squyres and Kasting, 1994). Remote (Mumma *et al.*, 2009) and satellite (Formisano *et al.*, 2004) observations have detected the presence of methane in the martian atmosphere, and results from atmospheric sampling by the Curiosity rover imply the irregular presence of a plume of methane (Webster *et al.*, 2015). Although an abiogenic source of methane on Mars requires serious consideration, its origin from presently active biology remains a possibility (Court and Sephton, 2009).

A future Mars Sample Return (MSR) mission would aim to utilize the unrestricted investigative potential of Earth-based instrument suites to provide further insight into the question of martian life (McLennan *et al.*, 2012). The requirements of the MSR *in situ* mission phase would differ from those on previous Mars missions. MSR *in situ* operations would attempt to identify and cache samples that exhibit high scientific potential, rather than seeking maximum scientific return while on the martian surface.

For the analysis of rock samples *in situ* on Mars, previous missions have generally employed mass spectroscopy or gas chromatography–mass spectrometry (GC-MS) instrument concepts (Viking, Beagle 2, Phoenix, and Curiosity) (Klein *et al.*, 1976; Sims *et al.*, 2000; Hoffman *et al.*, 2008; Mahaffy *et al.*, 2009). GC-MS provides characterization and quantification of molecular species and can be sensitive to low organic contents. If similar results to those provided by GC-MS can be achieved but at lower operational costs (mass, power, materials), then sample triage for MSR would be facilitated, and assessment of a higher number of samples possible.

Fourier infrared spectroscopy (FTIR), which has been used on a number of space missions, provides a significant investigative return at low operational costs. FTIR is a relatively rapid and simple technique that delivers a large amount of information about the chemical nature of the analytes. To date, FTIR instruments have been used on board robotic Mars landers for remote sensing of rocks, though it has been proposed that use of FTIR could be extended to drilled samples (Anderson *et al.*, 2005). FTIR instruments have generally lower sensitivities, when compared to GC-MS, but the fewer resource and analytical requirements make FTIR attractive for screening and caching samples for future return to Earth.

A pyrolysis-FTIR instrument has been proposed to fulfill the role of triage for a MSR mission (Sephton *et al.*, 2013). Pyrolysis-FTIR involves heating solid samples rapidly (up to 20,000°C s^−1^) to liberate gaseous products that are subsequently characterized and quantified with IR transmission spectra. A pyrolysis-FTIR instrument can comfortably meet the weight, power, and structural requirements of a mission landed on the martian surface, as the primary components involved have all been successfully used on a number of missions. For example, pyrolysis ovens were used on the Mars Science Laboratory (MSL) mission (Mahaffy, 2008), and remote scanning FTIR spectrometers were employed on the Mars Exploration Rover mission (Christensen *et al.*, 2003).

A sample should only be considered high priority for biosignature detection if it is known to come from a habitable environment. It has been demonstrated that pyrolysis-FTIR can provide diagnostic insight into the mineralogy of samples through which past habitability can be assessed (Gordon and Sephton, 2016). Hydrated and evaporite minerals reveal past aqueous conditions that are vital for habitability, and these can be identified by the temperature release profiles of gases in pyrolysis-FTIR analyses. The temperature at which water is liberated from a sample indicates the nature of the minerals in a rock, with higher temperatures required to liberate mineral-bound hydroxyls of serpentinites, for example, than the temperatures required for the release of adsorbed water from rock materials weathered at low temperatures. Carbonates, which mostly form in regions chemically and energetically favorable for life, are generally detectable through strong carbon dioxide signals from pyrolysis-FTIR analysis.

Once past habitability is established, the preservation potential for biosignatures must be considered (*e.g.*, Summons *et al.*, 2011). The stability of any biosignature is dependent on its initial form, the matrix in which it is hosted, and the chemical and physical processes it was subjected to over time. Some of the most favorable host rocks within which to capture microbial biosignatures are those rich in clay minerals and carbonates. It has been shown that pyrolysis-FTIR can be successful in detecting clay-rich sediments and carbonates (Gordon and Sephton, 2016), which, based on evidence from Earth's geological record, can maintain fossils for up to 3.5 × 10^8^ years (Farmer and Des Marais, 1999). Sulfates, also detectable by pyrolysis-FTIR through the production of sulfur dioxide, can offer fossil stabilities for timescales of up to 1 × 10^6^ years (Farmer and Des Marais, 1999). Thus, based on the presence of minerals known to preserve biosignatures, their preservation potential can be inferred from pyrolysis-FTIR results.

Because entirely abiotic mechanisms are able to form structures with cell-like morphologies similar to the biosynthetic structures of microbial cells, correlative organic compound detection is required before a case for life can be conclusively made. Organic compounds produce distinctive signals in the IR region, and gas-phase FTIR currently offers sensitivities on the order of a few parts per million. To date, the detection of organic compounds on the surface of Mars has proven difficult. The most abundant organic compounds detected were found at a few parts per billion in the Sheepbed Mudstone at Gale Crater by MSL (Freissinet *et al.*, 2015). The detection of organic molecules by a pyrolysis-FTIR instrument at concentrations of a few parts per million or above would indicate conditions conducive to the creation and preservation of organic matter. Any sample rich in organic matter would be of high scientific value; thus such a discovery during triage would be enough to select it for return to Earth.

This study aims to characterize the ability of a pyrolysis-FTIR instrument to detect organic matter in Mars samples. An assessment of various concentrations of organic matter in a mineral matrix provides information on the sensitivity limits of the instrument, while a survey of comparable concentrations across a number of Mars-relevant minerals aims to provide information on the effects of mineral matrices on the detection of organic matter.

## 2. Methods

### 2.1. Sample selection

This study required a suitable test biomaterial and a range of appropriate mineral matrices. *Lycopodium* spores powder, made from the dry spores of clubmosses, is available in commercially processed quantities and manageable for mixing with powdered minerals. Although the spores are the product of organisms more highly evolved than anything likely to have existed on Mars, they represent a reliable and reproducible assemblage of organic constituents.

Characterization of the response of this biomaterial allows comparison between modes of pyrolysis-FTIR operation and serves as a reference point for mineral effects. To achieve this, a mixture of F-110 quartz sand (U.S. Silica Company, 2016) and high-purity silica powder #31624 (Sigma-Aldrich, 2016) was produced at a ratio of 3:1, hereafter referred to as “quartz,” which allowed effective mixing and suspension of the *Lycopodium* spores. The choice of other mineral matrices was informed by the predominant eras of mineral alteration on Mars, as identified by Bibring *et al.* (2006); a serpentinite was chosen to represent the Phyllocian, a jarositic clay was selected for the Theiikian, and two palagonites (altered basaltic glass) were chosen for the Siderikian. Details of the materials used in this study are listed in Table 1 and detailed chemical composition presented in Appendix A1.

**Table 1.** Materials Used in This Study (See Appendix A1 for More Detailed Mineralogical Characterization)

*Role*	*Name*	*Source*	*Details*
Biomaterial	*Lycopodium*	Sigma-Aldrich	
Inert substrate	Quartz	Sigma-Aldrich/U.S. Silica	A mixture of three parts F-110 U.S. Silica quartz sand and one part high-purity silicon oxide powder from Sigma-Aldrich.
Phyllocian analogue	Serpentinite	Partially serpentinized peridotite, Kennack Sands, Cornwall, UK	[Lower-Mid Devonian] Regions on Mars which exhibit the minerals that characterize the Phyllocian era serve as the best candidates for past habitation.
Theiikian analogue	Jarositic clay	Parkstone Clay Member, Brownsea Island, Dorset, UK	[Eocene] Pyrite exposed to present-day atmosphere oxidizes to jarosite, a sulfur-rich mineral. Similar hydrated sulfates present on Mars are used to define the Theiikian era.
Siderikian analogue	Palagonitic tuff	Madeira, Portugal	[Pleistocene] Contains chlorinated phases. Chlorinated compounds influence the production of organic species during pyrolysis.
	JSC Mars-1	Pu'u Nene, Hawaii	[Holocene] Well-documented martian regolith simulant developed by NASA's Johnson Space Center (Allen *et al.*, 1998).

All minerals were powdered and homogenized using a pestle and mortar. *Lycopodium* spores were added and the total weight monitored through iterative mass measurements on a balance accurate to 0.1 mg to produce a 5% mixture by mass (an organic matter concentration typical of topsoils on Earth, thus chosen as a “best case” scenario starting point). Subsequent lower concentrations of 1% and 0.5% were produced, each made from a dilution of the former mixture. In the case of quartz, further mixtures were made for the purpose of sensitivity appraisal, from 0.45% to 0.05% (in 0.05% steps) and one at 0.02%. Each mixture was stored in a screw-capped vial and mixed manually for 5 min, to ensure homogeneity. It is recognized that the simple mixing process is not wholly representative of the juxtaposition and chemical bonding of organic matter and minerals that have undergone co-deposition and diagenesis, but the assumption was made that the mineral–*Lycopodium* spores mixtures adequately reflected the thermally induced processes experienced during pyrolysis-FTIR analysis of natural samples.

### 2.2. Pyrolysis-FTIR

Samples were loaded in quantities ranging from 7.5 mg to 23.4 mg into quartz tubes and held in place by quartz wool plugs at either end. Where it is deemed useful, a value of 15 mg is adopted as the representative pyrolysis-FTIR sample mass. Pyrolysis was achieved with a CDS Analytical Pyroprobe 5200, and the FTIR spectra were collected using a Thermo Scientific Nicolet 5700 FTIR spectrometer. Gases resulting from pyrolysis were contained within a CDS Brill Cell fitted with IR-transparent ZnSe windows. This gastight chamber was supplied with a controllable helium flow, which was used to maintain an oxygen-free atmosphere during analysis and purge the cell of any spent analytes. Both pyrolysis and FTIR operations were controlled by CDS 5000 DCI software and Thermo Scientific OMNIC Series software, respectively, the latter also being used to record and process spectra. Each session of sample analysis was preceded by collection of three or more spectra from procedural blanks. Unless otherwise stated, sample spectra are composed of 32 scans taken over 19.5 s at a resolution of 4 cm^−1^, collected immediately after the pyrolysis probe had ceased firing.

### 2.3. Sensitivity appraisal

To conduct the sensitivity appraisal, the optimal pyrolysis temperature for maximum signal response from *Lycopodium* spores was identified. First, pure quantities of *Lycopodium* spores were subjected to various heating rates, and the IR responses for organic structures were recorded as a function of time. These initial investigations were then corroborated with data from a stepped analysis. The acquired data indicate that most *Lycopodium* spores break down at or below 700°C. To test *Lycopodium* spore performance in a mixture and examine the influence of continued probe heating on the pyrolysis products, samples of 5% *Lycopodium* spores in quartz were subjected to a range of static temperatures, and the IR responses of the products were measured over a 30 s period. The data show that an analysis at 700°C and for 7.2 s produced the greatest yield of organic products, and this pyrolysis mode was used for all samples unless stated otherwise.

For a statistical investigation of sensitivity of pyrolysis-FTIR, a large number of data were required. A different number of data points were obtained for the various concentrations of *Lycopodium* spores studied: for 0.02%, 0.05%, and 0.10% (30 data points); for 0.15%, 0.20%, and 0.25% (15 data points); and for 0.30%, 0.35%, 0.40%, 0.45%, and 0.50% (3 data points).

Using the OMNIC Software Suite, the final spectra were generated by combining three individual blank-corrected spectra. Spectra were then truncated, leaving the 3300–2650 cm^−1^ wavenumber region, and the baseline was corrected to provide a baseline against which the total area under peaks in the C-H stretching region (3150–2740 cm^−1^) was recorded. Consideration of the total area in a relatively wide section of the frequency domain, rather than targeting specific peaks, allows for blanket detection of molecular structures containing C-H bonds. The signal produced by this method is hereafter referred to as the “hydrocarbon response.”

From sample mass measurements, the absolute quantity of *Lycopodium* spores in each sample was ascertained and plotted against the associated hydrocarbon response. A sensitivity analysis was performed by using the hydrocarbon responses. Results were grouped based on the mass of *Lycopodium* spores present in the sample (*m*), with limits chosen so that there was an adequate number of samples (*n*) in each grouping. The following limits were used: 1 μg ≤ *m* but *<* 5 μg (*n* = 30), 5 μg ≤ *m* but <10 μg (*n* = 29), 10 μg ≤ *m* but < 15 μg (*n* = 23), 15 μg ≤ *m* but < 25 μg (*n* = 29), *m* ≥ 25 μg (*n* = 39).

Sensitivity of the instrument (*i.e.*, the ability for the instrument to make a correct detection of organic matter) was investigated by calculating the rate of true positive detections as the detection threshold varied, defined as follows:

\begin{align*}
{\rm {sensitivity}} =   {\frac {\rm {number \ of \ true \
positives}} {\rm {number \ of \ true \ positives + number \ of \
false \ negatives}}}
\end{align*}

A result was deemed positive if the hydrocarbon response equaled or exceeded a chosen detection threshold, which in this case was multiples of the baseline fluctuation of the instrument (measured by taking the standard deviation of signals, in the same hydrocarbon response region, obtained from 35 procedural blanks).

Measurements of the hydrocarbon response from 15 pure quartz samples was used to investigate the specificity of the instrument, defined as

\begin{align*}
{\rm {specificity}} = {\frac {\rm {number \ of \ true \
negatives}} {\rm {number \ of \ true \ negatives + number \ of \
false \ positives}}}
\end{align*}

where a true negative would be a pure quartz sample producing a signal in the hydrocarbon region lower than the detection threshold (as described for sensitivity).

Values from both sensitivity and specificity investigations were plotted as a function of multiples of the same measure of the baseline fluctuation.

### 2.4. Assessment of mineral matrix effects

To assess the effects of minerals on pyrolysis-FTIR organic matter responses, two temperatures were used: 700°C, which recorded the optimal yield of hydrocarbons established by experimentation, and 1000°C, which was determined to be a temperature that led to high-sensitivity detection for mineral indicators of habitability (Gordon and Sephton, 2016). Three concentrations of the mineral–*Lycopodium* spore mixture were analyzed for each mineral type (5%, 1%, and 0.5%) in addition to the pure mineral (*i.e.*, 0%). Final spectra for each sample concentration were generated by combining three individual blank-corrected spectra.

Spectra were truncated to a 4000–1250 cm^−1^ wavenumber region with the OMNIC Software Suite and then baseline corrected. Peak intensities were then measured and recorded: one located at 2349 cm^−1^ corresponding to the anti-symmetric stretch in carbon dioxide; one at 3853 cm^−1^ arising from a stretching mode of water; one at 1352 cm^−1^ corresponding to the sulfur dioxide anti-symmetric stretching mode; one at 3016 cm^−1^ corresponding to the methane anti-symmetric stretching mode and the height of a peak at 2933 cm^−1^, which was present and dominant when pure *Lycopodium* spore samples were pyrolyzed.

Note that, if present, hydrogen chloride produced a fringe that overlaps the 3016 cm^−1^ peak used to measure the methane response. To overcome this overlapping issue, a reference spectrum for hydrogen chloride was obtained from the NIST database and subtracted from the sample spectra found to contain a hydrogen chloride signal, thereby allowing measurement of any methane peak. When applicable, the relative intensity of the 2798 cm^−1^ peak of hydrogen chloride was measured to account semi-quantitatively for the amount of hydrogen chloride produced. Table 2 lists the spectral features utilized in this study and their wavenumbers.

**Table 2.** Spectral Features Analyzed in Pyrolysis-FTIR Spectra to Measure Abundances of Gas Species of Interest

*Species*	*Vibrational mode*	*Wavenumber (cm*^−1^*)*
Carbon dioxide	Antisymmetric stretching	2349
Water	Stretching	3853
Sulfur dioxide	Antisymmetric stretching	1361
Methane	Antisymmetric stretching	3015
Organic compounds	C-H stretching	3150–2740
Hydrogen chloride	Stretching	2798

Once peak intensities in the samples were recorded, the equivalent responses taken from blanks (averaged for the relevant collection session) were subtracted, and the resulting values were averaged for each set of samples (*i.e.*, the three repetitions of each sample type were averaged). The resulting averaged peak values for carbon dioxide, water, sulfur dioxide, and methane could be translated to quantified masses by reference to mass calibration curves, and in turn, the values for masses of gas could be expressed as percentages of the mass of mineral in each sample. Expressing masses of gas relative to the mineral component allowed direct comparison of results across all concentrations.

## 3. Results

### 3.1. Sensitivity appraisal

**FIG. 1.**
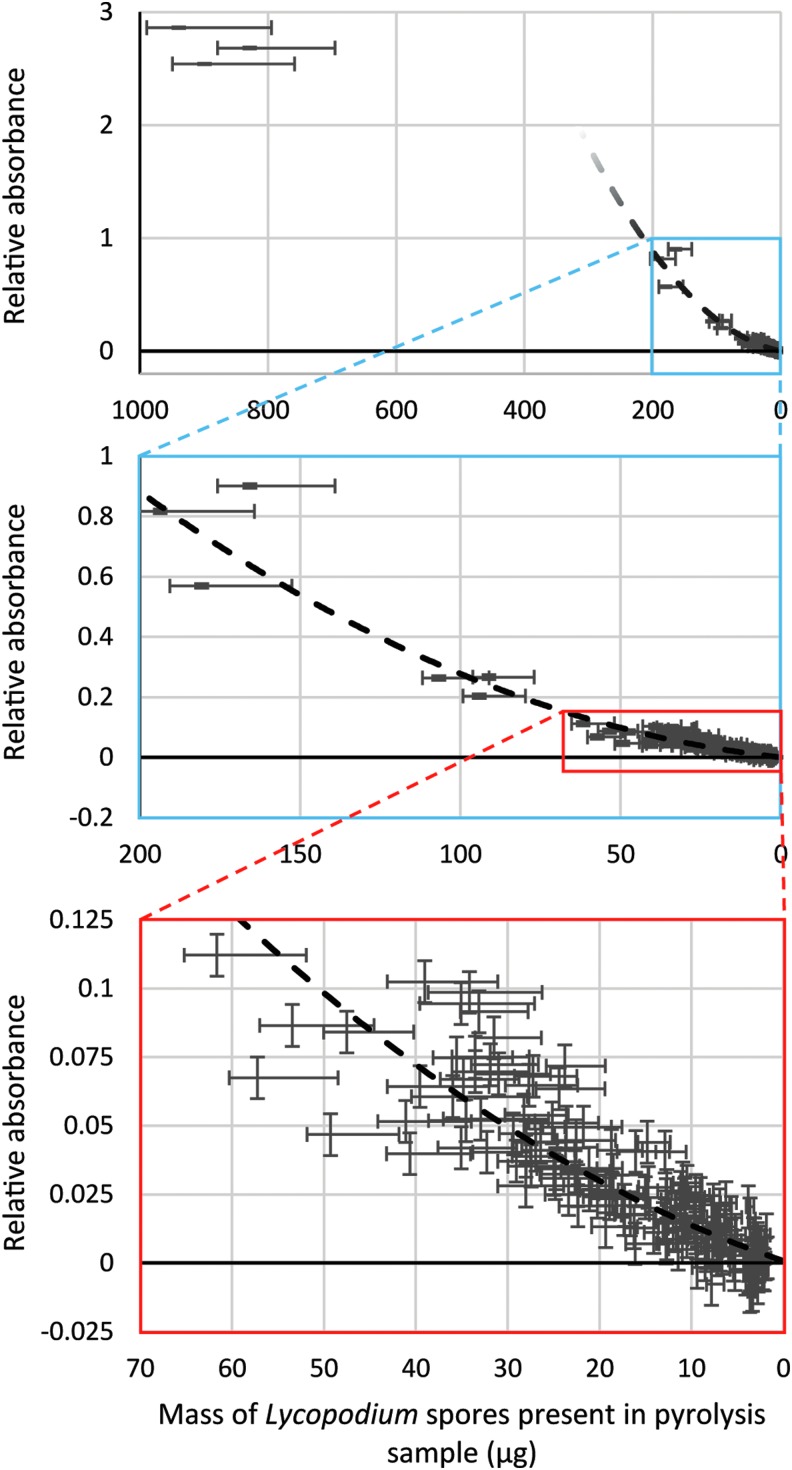
Absorbance (measured by taking the total area under the combined peaks) in the hydrocarbon stretching region (3150–2740 cm^−1^) plotted as a function of the quantity of *Lycopodium* spores present in the sample. All samples were subjected to 700°C for 7.2 s before the evolved gases were measured by FTIR. For this study, quartz served as an inert substrate and provided a baseline against which the additional results were compared. Vertical error bars represent one standard deviation of the data produced by procedural blanks, while horizontal error bars arise from the uncertainty in mass measurements, with additional consideration made for *Lycopodium* spore loss during sample handling (10% of expected mass) for the lower uncertainty boundary. The dashed trend line represents a best-fit quadratic function for data points with *Lycopodium* spore mass <200 μg, which is numerically represented as *A* = 2 × 10^−5^*m*^2^ + 0.0011*m* + 0.0008 with a coefficient of determination of 0.9301, where *A* is the relative absorbance and *m* is the mass of *Lycopodium* spores. Three high-concentration samples, with *Lycopodium* spore masses > 800 μg, do not adhere to this law, most likely due to saturation effects. It is interesting to note that the vertical intercept of 0.0008 is almost equal to the mean signal given by pure quartz samples of 0.0009 (*i.e.*, when *Lycopodium* spore mass is zero).

The correlation between the amount of *Lycopodium* spores present in a sample and the measured hydrocarbon response during pyrolysis-FTIR analysis was plotted as shown in Fig. 1. The responses were produced by taking the total area under overlapping absorbance peaks in the 3180–2680 cm^−1^ wavenumber region. The data points were used to produce a best-fit trend line shown in Fig. 1. A quadratic relationship described by the function *A* = 2 × 10^−5^*m*^2^ + 0.0011*m* + 0.0008 with a coefficient of determination of 0.9301, where *A* is the relative absorbance and *m* is the mass of *Lycopodium* spores, was found to represent all the samples with <200 μg *Lycopodium* spores in a 15 mg sample (i.e. < 1.3 wt %). This modeled the behavior of the instrument for amounts of *Lycopodium* spores < 200 μg, including the completeness of *Lycopodium* spore conversion to detectable compounds by pyrolysis, the efficiency of pyrolysate delivery to the gas containment cell and its residence time within, and the performance of the beam and detector system. Figure 1 therefore provided a calibration curve, and the strength of any detected absorbance signal area in the hydrocarbon region less than 1 could be referenced with the curve to determine the mass of organic material pyrolyzed.

It should be noted that the quadratic best-fit function only held true for amounts of *Lycopodium* spores that were <200 μg. The three 5% samples, which had *Lycopodium* spore quantities of >200 μg and did not follow the quadratic best-fit function for lower concentrations, most likely reflect saturation effects within the sampling gas cell owing to the large quantity of *Lycopodium* spores analyzed. Such saturation effects are relatively unimportant for this study because of the focus on low-sensitivity behavior. In Figure 1 it is apparent that the spread of data points lacks a certain degree of closeness to the trend line, which is likely due to uncertainty in the amount of *Lycopodium* spores present in the sample. *Lycopodium* spores readily become airborne as a consequence of their evolutionary design. Hence, *Lycopodium* spore losses might be expected during the sequential mixing steps and pyrolysis sample preparation, and unfortunately, such losses could not be readily quantified. As a result, detection limits determined by this study were nominal and serve as an upper boundary to the true limits (*i.e.*, they are a worst-case scenario).

**FIG. 2.**
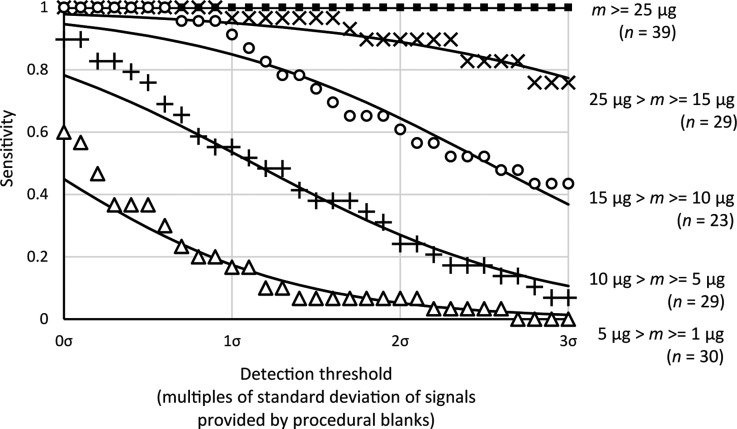
For a chosen detection threshold, there is a probability that pyrolysis-FTIR will make a detection given *Lycopodium* spores were present in sufficient quantities in a 15 mg sample. This figure shows how this probability (sensitivity) varies as a function of the chosen detection limit (multiples of the standard deviation obtained from responses in blanks) for a range of concentrations of *Lycopodium* spores–quartz mixture. The solid lines represent best-fit cumulative probability functions, which have be superimposed upon data points calculated from measurements.

The results of the sensitivity assessment are plotted in Fig. 2, where data points represent the probability of detection when an amount of *Lycopodium* spores is present in a given range. It can be seen that the data exhibit relationships where the sensitivity decreases as an increased detection threshold is imposed. The relationships between detection threshold and sensitivity have been identified to resemble cumulative probability functions, and these have been approximated with cumulative logistic distribution functions, given by

\begin{align*}
S\left(t\right) = {\frac {1} { 1 + e^{s \left( t - t_{1/2}
\right)}}}
\end{align*}

where *S* is the probability of making a positive organic compound detection (sensitivity), *t* is the detection threshold, *t*_½_ is the threshold value that gives *S* = 0.5, and *s* is a scaling parameter. The values for these parameters are listed for the different groupings of *Lycopodium* spore quantities in Table 3, and the relationships have been illustrated by solid lines in Fig. 2.

**Table 3.** Parameters for Cumulative Logistic Probability Functions Used to Model the Results of the Sensitivity Investigation

	n	t_½_	s	R^*2*^
*m* ≥ 25	39	—	—	—
25 > *m* ≥ 15	29	4.44	0.85	0.47
15 > *m* ≥ 10	23	2.52	1.13	0.85
10 > *m* ≥ 5	29	1.13	1.14	0.83
5 > *m* ≥ 1	30	−0.15	1.35	0.91

Using the relationship $ S\left(t\right) = {\frac {1} { 1 + e^{s \left( t - t_{1/2}
\right)}}}$ the probability of making a positive organic compound detection (or sensitivity *S*) can be calculated for a given detection threshold *t*, where *t*_½_ is the threshold value that gives *S* = 0.5 and *s* is a scaling parameter. *n* is the number of samples used to make each grouping.

The data suggest that when signals are required to be statistically significant (*i.e.* > 2σ), for a 15 mg sample only amounts of *Lycopodium* spores > 25 μg (0.17 wt %) will guarantee detection, and the probability of detecting an amount of *Lycopodium* spores < 5 μg (0.03 wt %) is ≈5%.

**FIG. 3.**
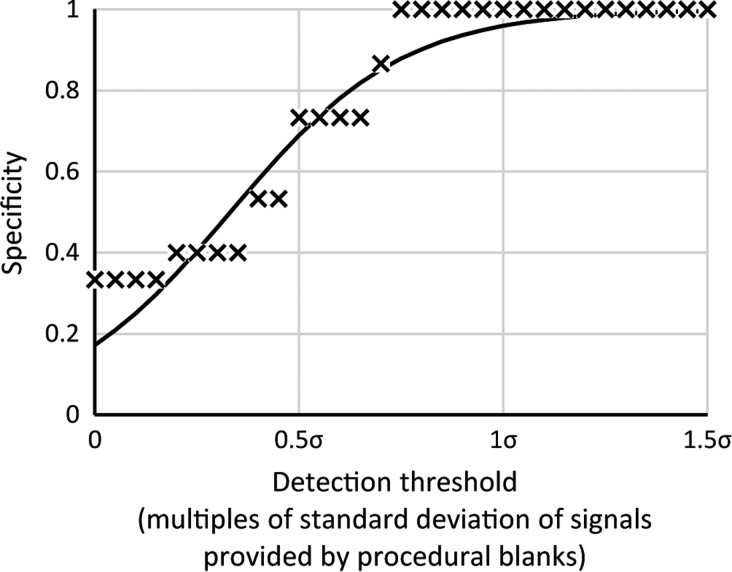
Specificity (also known as the true negative rate) of pyrolysis-FTIR organic compound detection as a function of detection threshold. The plot suggests that false positives are unlikely when a detection threshold greater than approximately one standard deviation of the baseline fluctuation is chosen.

The specificity was also investigated. Figure 3 shows the probability of a pyrolysis-FTIR analysis that would not produce a false positive as the required detection threshold was increased. The measured results were modeled with a cumulative logistic distribution as was done with the sensitivity data, with the parameters *s* = −4.73 and *t*_½_ = 0.33 (plotted as a solid line in Fig. 3, which had a coefficient of determination of 0.97). The results show that the likelihood of a false detection was virtually zero if a detection limit of >1σ was imposed. Reconciling this finding with the results of the sensitivity assessment, the probabilities of detection at 1σ for 15 mg samples containing *Lycopodium* spores are ≈17% for amounts 1 μg ≤ *m* but *<* 5 μg, ≈56% for amounts 5 μg ≤ *m* but <10 μg, ≈86% for amounts 10 μg ≤ *m* but <15 μg, ≈ 94% for amounts 15 μg ≤ *m* but < 25 μg, and 100% for amounts *m* ≥ 25 μg.

### 3.2. Mineral matrix effects

**FIG. 4.**
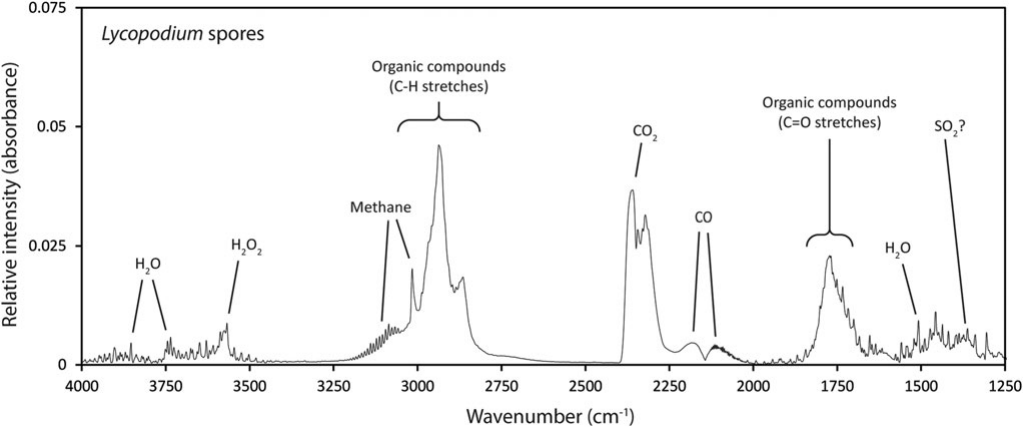
Pyrolysis-FTIR spectrum of pure *Lycopodium* spore powder with prominent features of interest labeled (pyrolyzed at 750°C).

**FIG. 5.**
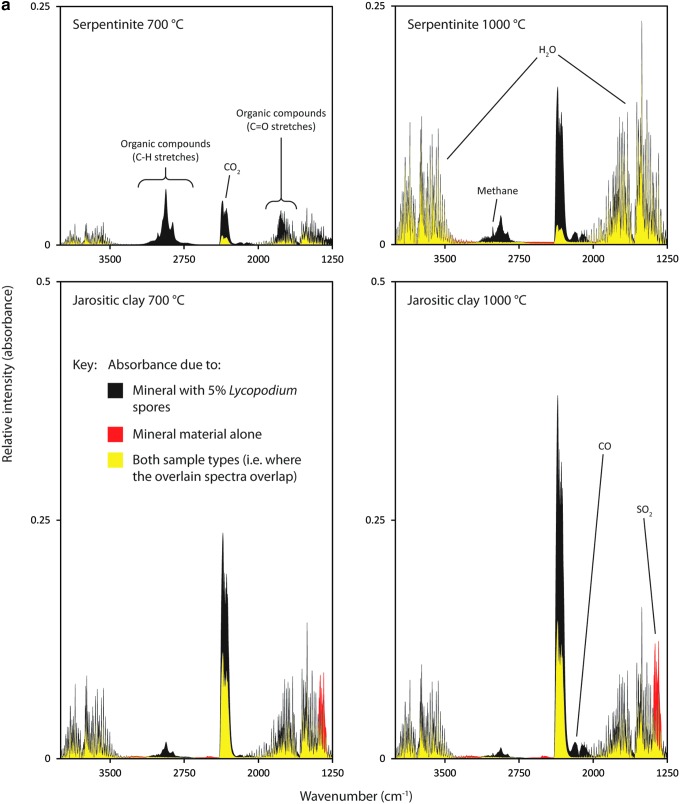

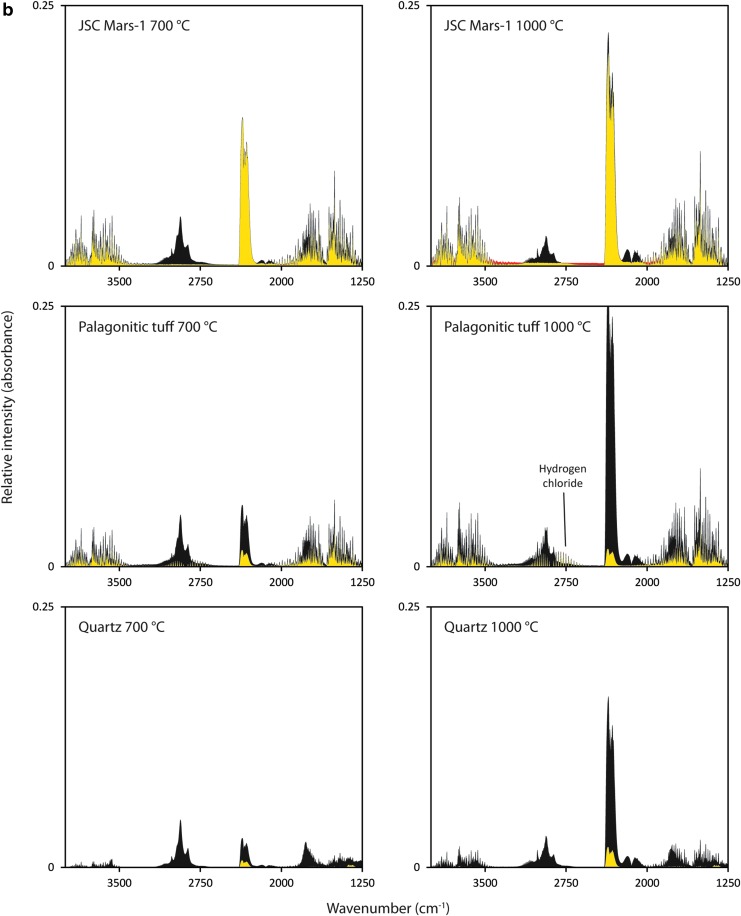
(**a**) Representative pyrolysis-FTIR results from serpentinite and jarositic clay, where the results of using the mineral material alone have been overlain with spectra produced by a mixture that contained 5% *Lycopodium spores*. Results have been scaled to represent a scenario where the quantity of mineral was equal in each case. The spectral features indicative of different gases are labeled. Color coding highlights where the presence of *Lycopodium* produces a surplus of a gas (black) or a deficit (red) when compared to the pure mineral samples. (**b**) Representative pyrolysis-FTIR results from JSC Mars-1, palagonitic tuff, and quartz, where the results of using the mineral material alone have been overlain with spectra produced by a mixture containing 5% *Lycopodium spores*.

A pyrolysis-FTIR spectrum of the *Lycopodium* spores is shown in Fig. 4. Representative spectra (formed from the average of three individual samples) of mineral and *Lycopodium* spore mixtures are displayed in Fig. 5a and Fig. 5b. These spectra have been color-coded to show where the presence of *Lycopodium* spores in a mineral matrix produces a greater (black) or lesser (red) response for particular gases when compared to an analysis of just the pure mineral. Quantitative data for the responses of carbon dioxide, water, sulfur dioxide, and methane are presented in Tables 4 and 5 (as percentages of the initial sample mass for the minerals without organic contents, and differences owing to *Lycopodium* spores in mineral mixtures as a percentage of the mass of the mineral components). Table 6 lists the relative intensities of absorbance in the wavenumber region associated with hydrocarbons, providing a semi-quantitative record of the organic matter pyrolysis products.

**Table 4.** Results from Pyrolysis-FTIR Survey at 700°C, Showing Quantities of Gases Produced from Pure Samples sans the Spores and the Differences Produced when *Lycopodium* Spores Were Introduced

	*wt %*			
	*CO_2_*	*Water*	*SO_2_*	*Methane*
*Quartz*				
	**0.058 ± 0.018**	**0.072 ± 0.015**	**0.043 ± 0.013**	**0.00085 ± 0.00016**
5.0%	0.20 ± 0.06	0.34 ± 0.09	0.00 ± 0.02	0.041 ± 0.006
1.0%	0.21 ± 0.06	0.16 ± 0.06	0.00 ± 0.02	0.010 ± 0.003
0.5%	0.16 ± 0.06	0.10 ± 0.04	0.005 ± 0.019	0.0023 ± 0.0008
*Serpentinite*				
	**0.083 ± 0.013**	**1.18 ± 0.15**	**−0.006 ± 0.0009**	**0.0001 ± 0.0002**
5.0%	0.35 ± 0.09	0.5 ± 0.5	0.043 ± 0.006	0.048 ± 0.007
1.0%	0.32 ± 0.10	0.30 ± 0.4	0.0110 ± 0.0019	0.0090 ± 0.0011
0.5%	0.22 ± 0.08	0.0 ± 0.5	0.0055 ± 0.0013	0.0040 ± 0.0007
*Jarositic clay*				
	**1.2 ± 0.2**	**4.0 ± 0.6**	**1.4 ± 0.4**	**−0.0022 ± 0.0005**
5.0%	1.9 ± 0.6	3.2 ± 1.5	−1.3 ± 0.4	0.0047 ± 0.0011
1.0%	1.4 ± 0.6	0.7 ± 1.2	−0.7 ± 0.5	0.0006 ± 0.0008
0.5%	0.8 ± 0.6	0.5 ± 1.2	−0.3 ± 0.6	0.0006 ± 0.0009
*Palagonitic tuff*				
	**0.14 ± 0.02**	**1.7 ± 0.2**	**0.009 ± 0.002**	**0.004 ± 0.002**
5.0%	0.42 ± 0.16	1.3 ± 0.9	0.042 ± 0.012	0.027 ± 0.008
1.0%	0.18 ± 0.08	0.5 ± 0.6	0.031 ± 0.011	−0.002 ± 0.003
0.5%	0.22 ± 0.11	0.3 ± 0.6	0.050 ± 0.014	−0.003 ± 0.003
*JSC Mars-1*				
	**1.6 ± 0.3**	**3.0 ± 0.5**	**−0.009 ± 0.003**	**0.0029 ± 0.0007**
5.0%	0.0 ± 0.7	1.1 ± 1.6	0.035 ± 0.012	0.045 ± 0.014
1.0%	0.3 ± 0.8	0.8 ± 1.5	0.005 ± 0.006	0.010 ± 0.004
0.5%	0.0 ± 1.0	0 ± 2	−0.003 ± 0.011	0.004 ± 0.004

Values in bold are responses from the pure mineral while the values in plain text indicate differences in the mass ratios when measured for samples where quantities of *Lycopodium* spores have been added (*i.e.*, a negative value represents a lower mass of a particular gas being produced in the mixture relative to the pure mineral). Three concentrations (5.0%, 1.0%, and 0.5%) of mineral–*Lycopodium* spore mixture were analyzed for each mineral matrix.

**Table 5.** Results from Pyrolysis-FTIR Survey at 1000°C, Showing Quantities of Gases Produced from Pure Mineral Samples sans the Spores and the Differences Produced When *Lycopodium* Spores Were Introduced

	*wt %*			
	*CO_2_*	*Water*	*SO_2_*	*Methane*
*Quartz*				
	**0.18 ± 0.03**	**0.063 ± 0.019**	**0.036 ± 0.014**	**−0.0002 ± 0.0002**
5.0%	1.7 ± 0.9	1.1 ± 0.3	0.00 ± 0.03	0.090 ± 0.019
1.0%	0.8 ± 0.2	0.38 ± 0.10	−0.015 ± 0.019	0.016 ± 0.003
0.5%	0.45 ± 0.17	0.21 ± 0.06	−0.01 ± 0.02	0.008 ± 0.002
*Serpentinite*				
	**0.17 ± 0.03**	**12 ± 2**	**−0.062 ± 0.010**	**−0.0037 ± 0.0011**
5.0%	1.8 ± 1.0	2 ± 5	0.026 ± 0.017	0.094 ± 0.018
1.0%	1.0 ± 0.4	−4 ± 6	0.03 ± 0.03	0.016 ± 0.004
0.5%	0.41 ± 0.19	−5 ± 6	0.03 ± 0.03	0.010 ± 0.004
*Jarositic clay*				
	**1.68 ± 0.17**	**4.8 ± 0.5**	**2.0 ± 0.2**	**−0.0022 ± 0.0004**
5.0%	4.3 ± 1.4	2.9 ± 1.7	−2.0 ± 0.2	0.023 ± 0.007
1.0%	2.4 ± 1.1	0.5 ± 1.4	−1.2 ± 0.3	0.0003 ± 0.0009
0.5%	1.5 ± 0.6	0.6 ± 1.2	−0.1 ± 0.4	−0.0004 ± 0.0008
*Palagonitic tuff*				
	**0.15 ± 0.05**	**1.7 ± 0.3**	**0.057 ± 0.018**	**0.0008 ± 0.0007**
5.0%	3.9 ± 1.6	2.7 ± 1.6	0.03 ± 0.04	0.08 ± 0.03
1.0%	1.5 ± 0.5	0.9 ± 1.0	0.04 ± 0.05	0.004 ± 0.002
0.5%	1.29 ± 0.38	0.5 ± 0.8	0.07 ± 0.06	0.0014 ± 0.0014
*JSC Mars-1*				
	**2.6 ± 0.6**	**3.5 ± 0.8**	**0.0005 ± 0.0019**	**0.0029 ± 0.0013**
5.0%	0.3 ± 1.2	1 ± 2	0.011 ± 0.009	0.07 ± 0.02
1.0%	−0.0 ± 1.2	0.3 ± 1.7	−0.010 ± 0.004	0.010 ± 0.004
0.5%	−0.1 ± 1.6	0 ± 2	−0.011 ± 0.008	0.004 ± 0.005

Values in bold are responses from the pure mineral while the values in plain text indicate differences in the mass ratios when measured for samples where quantities of *Lycopodium* spores have been added (*i.e.*, a negative value represents a lower mass of a particular gas being produced in the mixture relative to the pure mineral). Three concentrations (5.0%, 1.0%, and 0.5%) of mineral–*Lycopodium* spore mixture were analyzed for each mineral.

**Table 6.** Relative Strengths of Hydrocarbon Responses for Different Concentrations of *Lycopodium* Spore–Mineral Mixtures and Mineral Samples Free of *Lycopodium* Spores (Highlighted in Bold) from Pyrolysis-FTIR Analyses

	*Relative absorbance*	
	*700°C*	*1000°C*
*Quartz*		
	**0.00005 ± 0.00001**	**−0.00009 ± 0.00002**
5.0%	0.0448 ± 0.0018	0.029 ± 0.004
1.0%	0.0112 ± 0.0017	0.0051 ± 0.0003
0.5%	0.0034 ± 0.0003	0.00203 ± 0.00019
*Serpentinite*		
	**0.00004 ± 0.00001**	**0.000030 ± 0.000020**
5.0%	0.0572 ± 0.0015	0.0278 ± 0.0009
1.0%	0.0102 ± 0.0005	0.0039 ± 0.0007
0.5%	0.0030 ± 0.0008	0.00124 ± 0.00012
*Jarositic clay*		
	**−0.00027 ± 0.00005**	**−0.00059 ± 0.00006**
5.0%	0.0162 ± 0.0009	0.0107 ± 0.0009
1.0%	0.00011 ± 0.00004	−0.000056 ± 0.000010
0.5%	−0.00015 ± 0.00007	−0.00026 ± 0.00003
*Palagonitic tuff*		
	**0.00023 ± 0.00008**	**−0.000066 ± 0.000024**
5.0%	0.048 ± 0.004	0.0326 ± 0.0017
1.0%	0.00316 ± 0.00003	0.00080 ± 0.00006
0.5%	0.00097 ± 0.00004	0.00006 ± 0.00003
*JSC Mars-1*		
	**0.00026 ± 0.00004**	**0.00001 ± 0.00007**
5.0%	0.045 ± 0.003	0.027 ± 0.003
1.0%	0.0033 ± 0.0003	0.001630 ± 0.000014
0.5%	0.00131 ± 0.00016	0.00071 ± 0.00008

In this case, the response due to hydrocarbons is semi-quantitatively represented by the height of the dominant peak at the 2933 cm^−1^ wavenumber, found in the region strongly associated with the C-H stretches organic compounds (Fig. 4). Two temperatures of pyrolysis were compared in this study: 700°C and 1000°C.

In all cases, the production of hydrocarbons was evident with a few notable exceptions; jarositic clay and the palagonitic tuff appeared to produce no hydrocarbons at the 0.5% concentration at both 700°C and 1000°C, and produced no hydrocarbons at the 1.0% concentration at 1000°C. For jarositic clay and the palagonites, the production of hydrocarbons at 700°C was only observed in quantities that suggested rather than confirmed their presence.

In the following discussion, the term “mixtures” refers to the mineral in question to which some quantity of *Lycopodium* spores was added, and the term “pure” indicates the compound of interest without any added component (*i.e.*, minerals in the case of spores or spores in the case of minerals).

#### 3.2.1. *Lycopodium* spores (no minerals)

The pure *Lycopodium* spores produced a range of gases (Fig. 4). A significant amount of organic compounds was produced as was evidenced by the strong, broad, overlapping C-H stretching features at 2863 and 2937 cm^−1^, the methane peak at 3015 cm^−1^, and the strong, broad carbonyl group feature centered at 1776 cm^−1^. Water and carbon dioxide were also produced mainly as products of combustion, though some water was inherent in the natural constitution of the spores (up to 15% by weight, according to the manufacturer's description) that will be liberated by pyrolysis. Production of carbon monoxide was further evidence of combustion. *Lycopodium* spores produced a small feature at the region known for sulfur dioxide, but as this region was also shared with features characteristic of a number of organic functional groups (*e.g.*, alkane –C-H bending) and as the other organic features were so dominant, it was difficult to ascribe this feature conclusively to sulfur dioxide.

#### 3.2.2. Quartz

The pure quartz sample was found to be a useful inert substance against which other minerals could be compared. Contributions of carbon dioxide and sulfur dioxide were measurable following pyrolysis of pure quartz; however, the carbon dioxide amounts were small relative to amounts present in procedural blanks, and sulfur dioxide amounts were only approximately 450 ppm by weight. The carbon dioxide could be attributed to adsorbed species, while the sulfur dioxide could not have originated from the sample owing to its chemistry (Sigma-Aldrich, 2016; U.S. Silica Company, 2016) and is most likely a trace amount of carryover from previous experiments. Thus the quartz mixtures served to provide a relatively inert mineral matrix that provided insight into the flash pyrolysis breakdown of *Lycopodium* spores when suspended in the other minerals, yet the quartz sand mixture emulated the thermal and physical effects expected for mineral matrices with disbursed organic remains and thus offers a more representative standard material to enable comparisons than using pure *Lycopodium* spores.

For quartz and 5.0% *Lycopodium* spore mixtures at 700°C, responses were highly comparable to those for the pure *Lycopodium* spores. Even at the lowest concentration, all the *Lycopodium* spore indicators described in the previous section (and illustrated in Fig. 4) were present, which confirmed the quartz sand served as a suitable inert mineral matrix.

#### 3.2.3. Serpentinite

The pure serpentinite sample produced significant quantities of water upon pyrolysis. The water contribution was vastly increased at the higher 1000°C temperature step (shown clearly in Fig. 5a). The contribution of carbon dioxide was comparable to that seen in the pure quartz samples and thus was not introduced by the addition of serpentinite.

For the serpentine mixtures, methane results were in very close agreement with those seen for the quartz mixtures. For example, at 1000°C the *Lycopodium* spores in the serpentinite sample produced 0.090 ± 0.019, 0.016 ± 0.003, and 0.0078 ± 0.0019 wt % methane at 5.0%, 1.0%, and 0.5% concentrations of *Lycopodium* spores, respectively, while the equivalent quartz mixtures produced 0.090 ± 0.017, 0.013 ± 0.003, and 0.006 ± 0.003 wt % methane. The relative absorbance strengths in the hydrocarbon region were also concordant with those seen in the quartz mixtures (Table 6).

Thus the *Lycopodium* spores did not undergo any additional organic compound degradation relative to that observed in the quartz mixtures; hence the contribution of carbon dioxide from *Lycopodium* spores in serpentinite matches that produced by *Lycopodium* spores in quartz. Any variation in water released from *Lycopodium* spores in the presence of serpentinite was overwhelmed by the substantial signal from water released from serpentinite itself.

#### 3.2.4. Jarositic clay

The pure jarositic clay sample showed a substantial release of carbon dioxide, water, and sulfur dioxide at both temperatures, with the 700°C step producing these gases at approximately 70–80% of the quantities produced at 1000°C. The response of the jarositic clay sample was dramatically different to that of the serpentinite, where the pyrolysis of the latter at 700°C only liberated approximately 10% of the water produced at 1000°C. It was apparent that water was more readily released from the jarositic clay sample when subjected to pyrolysis-FTIR analysis. Weathered materials such as jarositic clay contain significant amounts of water and produce stronger water signals at lower temperatures than materials that are predominantly formed by the products of hydrothermal metamorphism represented by serpentinite.

When *Lycopodium* spores were introduced, a sharp drop in the sulfur dioxide signal was evident, and the reduced response corresponded to the quantity of *Lycopodium* spores present. Simultaneously, there was a sharp increase in the responses of water and carbon dioxide, and a reduction in hydrocarbon responses. It was clear that sulfur dioxide, produced from the sulfate content of the jarositic clay, was assisting the combustion of *Lycopodium* spore pyrolysis products by acting as a source of oxygen gas. The issue of sulfate compounds hindering the thermally assisted detection of organic matter on Mars has been highlighted (Lewis *et al.*, 2015). The production of sulfuric acid also presents another destruction mechanism for hydrocarbons, though the spectral features of sulfuric acid vapor (Hintze *et al.*, 2003) were not readily discernible in the spectra of the pure jarositic clay sample, as they coexist in regions obscured by the rotational-vibrational fringes of water.

At lower concentrations of *Lycopodium* spores in jarositic clay, both 0.5% and 1.0%, the hydrocarbon signal disappeared entirely for both 700°C and 1000°C temperature steps. The relative intensities of the carbon dioxide, water, and sulfur dioxide signals, however, relay information on the quantity of *Lycopodium* spores initially present. For the samples containing *Lycopodium* spore concentrations of 0.5% and 1.0%, it was observed that the extent to which the 1352 cm^−1^ sulfur dioxide peak diminishes is proportional to the quantity of spores present. The sulfur dioxide signal from the 0.5% *Lycopodium* spores in jarositic clay appeared to be slightly lower than for pure jarositic clay (although the decrease did not exceed the margins of error). The sulfur dioxide signal was measurably diminished (approximately 40% and 60% of the pure jarositic clay sulfur dioxide signal at 700°C and 1000°C, respectively) when the *Lycopodium* spore concentration was increased to 1.0%. For the 5.0% *Lycopodium* spores in jarositic clay, both 700°C and 1000°C results show that the sulfur dioxide peak was lost, suggesting that the entire budget of sulfur dioxide had been exhausted before the complete oxidative destruction of *Lycopodium* spore pyrolysis products could occur.

#### 3.2.5. Palagonitic tuff

The pure palagonitic tuff sample produced similar quantities of water at both 700°C and 1000°C, which indicated that the form of hydration was different to that of pure serpentinite, which generated more water at 1000°C than it did at 700°C. At 700°C the carbon dioxide produced from the palagonitic tuff sample was higher than that observed for the pure quartz sample at the same temperature. The carbon dioxide signal in pure palagonitic tuff at 1000°C, however, was comparable with that observed for pure quartz. The release of carbon dioxide at the lower temperature (700°C) was probably related to the liberation of adsorbed gas. The ability of palagonite to readily adsorb carbon dioxide has been recognized previously in Mars-related studies (Zent *et al.*, 1987).

A comparison of the gases released upon pyrolysis of the palagonite/spore mixtures showed that the palagonitic tuff mixtures produced only slightly increased amounts of carbon dioxide and water than were produced for the equivalent quartz mixtures at 700°C. The quantities of carbon dioxide and water generated by the palagonitic tuff mixtures, however, were significantly greater than that of the quartz mixtures at 1000°C. This finding suggests that a mechanism was present at 1000°C that was not substantial in its effects at 700°C and may be related to preexisting natural weathering products in the palagonite (Eggleton *et al.*, 1987) that were affected by pyrolysis at high temperatures.

The hydrogen chloride peaks observed in the palagonitic tuff starting material were diminished following the addition of *Lycopodium* spores (Table 7). Aside from the 5.0% samples and 1000°C temperature experiment, it was evident that the more *Lycopodium* spores present, the greater the reduction in the hydrogen chloride signal. Thermally assisted chlorination of *Lycopodium* spore organic matter is a plausible explanation for the features observed. Though the prevailing hypothesis that organic matter chlorination occurs during thermal extraction of Mars samples due to the presence of perchlorate minerals (Glavin *et al.*, 2013), the results of this study indicate that palagonite may also contribute to chlorination reactions.

**Table 7.** Relative Absorbance Responses for Hydrogen Chloride Produced from the Pure Palagonitic Tuff Sample and the Palagonitic Tuff Mixtures with *Lycopodium* Spores

		*700°C*	*1000°C*
*Lycopodium* spore concentration	0.0%	0.00581 ± 0.00010	0.0140 ± 0.0002
	5.0%	0.0027 ± 0.0003	0.0109 ± 0.0004
	1.0%	0.0033 ± 0.0003	0.0098 ± 0.0003
	0.5%	0.0040 ± 0.0005	0.0107 ± 0.0011

#### 3.2.6. JSC Mars-1

Figure 5b shows that the pure JSC Mars-1 sample acted as a source of carbon dioxide and water and that the production of these gases increased with temperature. In mixtures of *Lycopodium* spores and JSC Mars-1, the detected levels of organic compounds, methane, and carbon monoxide were comparable with levels for the same pyrolysis products in the quartz mixture experiments (see Fig. 5b), suggesting that *Lycopodium* spores were undergoing the same degradation processes in both mineral matrices (*i.e.*, JSC Mars-1 had no chemical influence on hydrocarbon degradation during pyrolysis-FTIR analyses). But unlike the case for quartz, the introduction of *Lycopodium* spores to JSC Mars-1 did not result in an increase in water and carbon dioxide at either 700°C or 1000°C. As a palagonitic tuff, JSC Mars-1 is a basaltic material that is highly susceptible to weathering by water and carbon dioxide mixtures (Eggleton *et al.*, 1987). Such weathering-type processes may be initiated and accelerated during thermal processing in the presence of water and carbon dioxide, leading to the reaction of these gases with the palagonitic minerals and their loss from the gas-phase pyrolysis products, at least at 700°C.

## 4. Discussion

For a rapid assessment of past habitability with multiple samples, a pyrolysis-FTIR method that can provide high sensitivity and diagnostic information is desired. A range of pyrolysis methods can be chosen, but for the temperatures examined in this study, the highest temperature (1000°C) produces the greatest response. A multistep approach, however, is most diagnostic, and during operation a choice must be made on the priorities for acquisition of either information type (Gordon and Sephton, 2016).

For the detection of an organic matter–containing sample on Mars, a pyrolysis-FTIR procedure that has the highest possible sensitivity is preferred. A range of pyrolysis temperatures are available, though for the spore-mineral mixtures for the two temperatures examined in this study, the lower temperature (700°C) produced the greatest organic matter response (Table 6). The higher-temperature pyrolysis-FTIR analysis at 1000°C was more sensitive to the common simple organic degradation products, namely methane, carbon dioxide, and water.

Figure 1 and the results of the sensitivity and specificity assessments revealed that the instrument in its current form only guarantees detection of total organic contents greater than 15 μg, and for a typical pyrolysis-FTIR sample mass of 15 mg, this equates to 1 part per thousand, a value significantly higher than required for effective triage on Mars. For example, MSL was tasked with finding organic matter in rocks at a few parts per billion in 0.078 cm^3^ of powdered sample, the maximum volume of material that can be delivered to its pyrolysis quartz cup when conducting evolved gas analysis (Mahaffy *et al.*, 2012). Given a plausible density of 3 g cm^−3^ for a serpentinite, and using a MSL equivalent sample size, the detection limit of the current version of our pyrolysis-FTIR system is 65 ppm. Extending this reasoning to the lower quantities of organic matter tested does not guarantee its detection: pyrolysis-FTIR has a ≈89% probability of identifying Mars samples with organic matter in the 64–43 ppm range, ≈56% in the 43–22 ppm range, and ≈17% in the 21–4 ppm range.

The juxtaposition of organic matter and some minerals during pyrolysis can result in the transformation (*e.g.*, chlorination) and destruction (*e.g.*, combustion) of organic matter, thereby hindering its direct detection. Again, the choice of temperature plays an important role in limiting the obfuscating effects of some minerals when attempting to detect organic signals. The lower pyrolysis temperatures promote fewer mineral-induced reactions than the higher pyrolysis temperatures.

As mineral effects inevitably depend on the mineral (or minerals) present during pyrolysis, analytical windows can be found where organic detection becomes possible. In the case of palagonitic tuff, where significantly less hydrogen chloride was produced at 700°C than at 1000°C, the detection of hydrocarbons was possible at the 1.0% concentration at the lower temperature. The results from jarositic clay showed that sulfate-bearing minerals posed a significant threat to the detection of organic compounds by thermal extraction methods, undermining the relatively high preservation potential associated with sulfates (Aubrey *et al.*, 2006). In the case of serpentinite, the mineral poses no real threat to the detection of organic material by thermal extraction methods, other than by masking the water signal produced by combusted hydrocarbons. Regions on Mars defined by relative abundances of hydrated minerals, like serpentinites, present the best locations for MSR sample acquisition when sample assessment is performed by pyrolysis-based methods.

Even for minerals that induce complete combustion of any organic matter present, the amount and temperature of release for carbon dioxide provide information relevant to the search for organic matter (Sephton *et al.*, 2014). Carbon dioxide is readily detectable by FTIR and can be recognized at a few parts per million or less for pyrolysis-FTIR. Thus any Mars rock sample subjected to pyrolysis-FTIR that produces a strong and correlatable carbon dioxide and water signal in a temperature range consistent with organic matter combustion and with no obvious mineral source or host for this gas could be considered high priority for biosignature detection and selected for return to Earth.

## 5. Conclusions

Pyrolysis-FTIR has the potential to be an information-dense method for Mars sample triage. A pyrolysis-FTIR instrument can produce true positives for organic compounds when they are present in quantities of tens of parts per million. In common with other thermal extraction-based methods, organic compounds are susceptible to mineral effects during pyrolysis-FTIR. Inevitably the particular type of mineral matrix determines whether organic compound transformation takes place. Chlorine- and sulfur-bearing minerals pose a threat to organic pyrolysis products, although the recognition of degradation products provides another avenue for detection. The iron-sulfate mineral jarosite, which is present on Mars, can cause the oxidation of organic products during pyrolysis. Palagonite is also common on the Red Planet and should be added to the list of mineraloid alteration products that could chlorinate organic compounds during thermal extraction. Selection of pyrolysis temperature is an important consideration since lower temperatures may limit the release of potential destructive agents from the host mineral matrix while retaining a range of organic compounds and degradation products that can be detected.

## Appendix A1

**Table:** (a) Mineralogical Analysis of Samples by X-ray Diffraction

*Sample*	*Illite + Mica*	*Kaolinite*	*Chlorite*	*Lizardite*	*Quartz*	*Plagioclase*	*Augite*	*Enstatite*
Serpentinite	0.0	0.0	0.0	43.8	3.0	0.0	0.0	14.8
Palagonite	0.0	0.0	0.0	0.0	3.5	63.7	25.7	0.0
Jarositic clay	7.1	8.2	0.0	0.0	44.7	0.0	0.0	0.0

* (continued)*								
*Sample*	*Forsterite*	*Amphibole*	*Halite*	*Jarosite*	*Magnetite*	*Goethite*	*Hematite*	*Total*
Serpentinite	28.6	0.0	0.0	0.0	4.9	0.0	4.9	100.0
Palagonite	0.0	trace	7.1	0.0	0.0	0.0	0.0	100.0
Jarositic clay	0.0	0.0	0.0	4.8	0.0	35.2	0.0	100.0

Quantified by Rietveld (Autoquan and SiroQuant). No samples contained illite/smectite or potassium feldspar.

**Table:** (b) Mineralogical Specification for Samples from U.S. Silica Company Material Safety Data Sheet (U.S. Silica Company, 2016)

*Sample*	*Quartz*	*Aluminium oxide*	*Iron oxide*	*Titanium oxide*
Quartz sand	99.0–99.9	<1.0	<0.1	<0.1

## Abbreviations Used

FTIR: Fourier infrared spectroscopy
GC-MS: gas chromatography–mass spectrometry
MSL: Mars Science Laboratory
MSR: Mars Sample Return
